# Uptake of HLA Alloantigens via CD89 and CD206 Does Not Enhance Antigen Presentation by Indirect Allorecognition

**DOI:** 10.1155/2016/4215684

**Published:** 2016-06-20

**Authors:** Eytan Breman, Jurjen M. Ruben, Kees L. Franken, Mirjam H. M. Heemskerk, Dave L. Roelen, Frans H. Claas, Cees van Kooten

**Affiliations:** ^1^Department of Nephrology, Leiden University Medical Center (LUMC), 2333 ZA Leiden, Netherlands; ^2^Department of Immunohematology and Blood Transfusion, LUMC, 2333 ZA Leiden, Netherlands; ^3^Department of Hematology, LUMC, 2333 ZA Leiden, Netherlands

## Abstract

In organ transplantation, alloantigens are taken up by antigen presenting cells and presented via the indirect pathway to T-cells which in turn can induce allograft rejection. Monitoring of these T-cells is of major importance; however no reliable assay is available to routinely monitor indirect allorecognition. Recently we showed that HLA monomers can be successfully used to monitor indirect allorecognition. Targeting antigens to endocytic receptors on antigen presenting cells may further enhance the presentation of antigens via HLA class II and improve the efficiency of this assay. In the current study we explored targeting of HLA monomers to either CD89 expressing monocytes or mannose receptor expressing dendritic cells. Monomer-antibody complexes were generated using biotin-labeled monomers and avidin labeling of the antibodies. We demonstrate that targeting the complexes to these receptors resulted in a dose-dependent HLA class II mediated presentation to a T-cell clone. The immune-complexes were efficiently taken up and presented to T-cells. However, the level of T-cell reactivity was similar to that when only exogenous antigen was added. We conclude that HLA-A2 monomers targeted for presentation through CD89 on monocytes or mannose receptor on dendritic cells lead to proper antigen presentation but do not enhance indirect allorecognition via HLA-DR.

## 1. Introduction

In organ transplantation CD4 T-cells can recognize HLA alloantigens either after internalization and processing by recipient “antigen presenting cells” (APC, indirect pathway) or directly on donor APCs (direct pathway) [1]. Experimental and clinical studies have demonstrated that indirect alloreactive T-cells are crucial for the formation of alloantibodies [1–3] and that these Abs are associated with reduced graft survival [4]. Furthermore, clinical studies have shown that indirect alloreactive CD4 T-cells are correlated with chronic rejection [5]. Although short-term allograft survival has increased dramatically over the past decades, long-term allograft survival has remained largely unchanged [6, 7]. It is therefore crucial to develop tools that enable monitoring of T-cell alloreactivity over time. Currently there is no reliable routine test available to measure indirect alloreactive CD4 T-cells in the clinic, although several attempts have been made [8]. Recently, we developed a method to monitor indirect allorecognition making use of HLA class I monomers [9]. However, the procedure requires relative high concentrations of monomer, associated with high costs, which is a serious drawback for the use of this system. We have therefore looked for strategies to improve antigen presentation.

Exogenous antigens are traditionally processed by endocytosis or pinocytosis and presented via HLA class II to CD4 T-cells [10], although they can also be presented in the context of HLA class I by cross-presentation to CD8 T-cells [11]. Preferential antigen targeting and presentation can be achieved through targeting of the antigens to endocytic receptors on APCs. APCs express multiple endocytic receptors which mediate transport of the antigens to endocytic compartments for processing and presentation [12].

Several endocytic receptors have been previously described as candidates for antigen specific targeting to HLA class II [13–16]. The IgA Fc receptor (Fc*α*RI/CD89) is highly expressed on monocytes, with only minimal expression on “monocyte-derived dendritic cells” (moDCs) [17, 18]. Targeting of* E. coli* to CD89 on monocytes has led to efficient bacterial uptake into these cells and a rapid breakdown of the bacteria [19]. Furthermore, targeting of ovalbumin to monocytes via CD89 led to trafficking of the antigenic cargo into HLA class II containing compartments and to the presentation of ovalbumin derived peptides via HLA class II to T-cells [15, 20, 21].

Another receptor frequently used for antigen targeting is the “mannose receptor” (MR/CD206), a C-type lectin receptor (CLR) not expressed on monocytes but highly expressed on DCs. The MR has been shown to mediate antigen uptake and presentation via HLA class II to CD4 T-cells [14, 22, 23]. The MR is an endocytic receptor that recognizes carbohydrate moieties, which is continuously recycled between the plasma membrane and the early endosomal compartment with its bound ligand [24]. The endosomal acidification induces ligand release and the empty receptor is recycled to the cell surface [25]. Recently the mannose receptor has also been implicated in the presentation of antigens to CD8+ T-cells in addition to CD4+ T-cells* in vitro* [26]. Furthermore,* in vivo* targeting of tumor antigens via MR has led to significant reduction in tumor sizes by inducing an increased antitumor immunity [27, 28].

In the current study we have investigated the possibility of CD89 and CD206 targeting on monocytes and moDCs to enhance processing of HLA class I alloantigen and antigen presentation to CD4 T-cells, as a tool to facilitate the detection and monitoring of indirect T-cell alloreactivity.

## 2. Materials and Methods

### 2.1. Cell Culture and Reagents

HLA typed (HLA-DR1+/HLA-A2−) buffy coats were obtained from the Dutch blood bank (Sanquin, the Netherlands). moDCs were differentiated from monocytes as previously described [29]. Briefly, monocytes were isolated using CD14 labeled magnetic beads (Miltenyi Biotec, the Netherlands) according to manufacturer's protocol. Monocytes were cultured for 6 days in RPMI-1640 (PAA, Austria) containing 10% FCS (Bodinco, the Netherlands) and 5,000 U/mL penicillin and 5 mg/mL streptomycin (both from invitrogen, USA) in the presence of 5 ng/mL GM-CSF and 10 ng/mL IL-4 (both Gibco, Invitrogen, USA). Cytokines were refreshed every 2-3 days.

HLA-A^*∗*^0201 derived peptides (20 amino-acids of length, region 98-118) were synthesized by solid phase peptide synthesis. Purification of peptides was confirmed by reverse-phase HPLC and by amino-acid analysis.

### 2.2. Antibodies and Flow Cytometry

Prior to staining, cells were pelleted and washed in phosphate buffered saline containing 0.02% sodium azide, 1% bovine serum albumin, and 1% heat inactivated normal human serum.

Monocytes and moDC were stained for HLA-DR (clone B8.11.2, IgG2b), HLA-A2 and CD14 (both from BD Biosciences), CD206/“mannose receptor” (MR, clone D547.3, IgG1 [30]), CD209/DC-SIGN (R&D, IgG2a), and CD89 (clone 2D11, IgG1 [31]). Staining was visualized with secondary antibodies, that is, goat anti-mouse Ig-F(ab)2-APC or goat anti-mouse Ig-F(ab)2-PE (both from Dako, Glostrup, Denmark). Mouse isotypes controls used were IgG1, IgG2a, and IgG2b (all from BD Biosciences). Staining was visualized by flow cytometry on a FACSCalibur equipped with CellQuest software (both from BD). Viable cells were gated based firstly on FSC/SSC and secondly using Annexin-V/PI staining kit (Molecular probes, Invitrogen, USA) to distinguish between viable and dead or apoptotic cells. In all experiments only viable cells were selected.

### 2.3. T-Cell Clone

A CD4+ T-cell clone recognizing an epitope from 98–120aa region of an HLA-A^*∗*^0201 in the context of HLA-DR1 was previously characterized and prepared as described [32]. Briefly, the T-cell clone was maintained in IMDM (Lonza) medium containing 100 IU/mL recombinant IL-2 (Chiron, Novartis, Emeryville, CA, USA), 5% FCS, 5% NHS (Sanquin, the Netherlands), 5,000 U/mL penicillin, and 5 mg/mL streptomycin. Specificity of the T-cell clone was routinely tested and validated.

### 2.4. Antibody Conjugation

Biotinylated recombinant HLA-A2 monomers (based on the HLA-A^*∗*^0201 sequence, containing a biotin tag in the *α*3 site) were created as previously described [33]. Briefly, HLA-A^*∗*^0201 heavy chains were produced in* Escherichia coli*. Biotinylated-monomer refolding was around the melanoma-associated pmel 17 peptide (YLEPGVTA) in the presence of *β*2-microglobulin. Monomers were purified by gel filtration HPLC, routinely tested, and validated.

Antibodies targeting the MR (CD206, clone D547.3) or Fc*α*RI receptor (CD89, clone 2D11) were conjugated to avidin using the LL-avidin kit (Innova biosciences, UK) according to manufacturer's protocol. “Avidinylated antibody” (referred to as CD206-A or CD89-A) specificity was confirmed by flow cytometry and compared with the non-avidinylated antibody (CD206 or CD89). An ELISA was set up to confirm the formation of the monomeric HLA-A2 and “antibody” (Ab) complex. MaxiSorp flat-bottomed 96-well plates (Nunc, Thermo Scientific) were coated with 1 *μ*g of CD206-A/CD206 or CD89-A/CD89 (diluted in PBS) and incubated for 2 hours at 37°C. After each incubation step plates were washed extensively (at least 5x) with PBS containing 0.05% Tween-20 and 1% BSA (both from Sigma-Aldrich). All subsequent incubation steps were for 1 h at 37°C. Different concentrations of biotinylated HLA-A2 monomer were added to the coated wells, followed by 1 *μ*g/mL of anti-HLA-A2 (IgG2b, BD Biosciences) acting as capture Ab. 1 *μ*g/mL of goat anti-mouse IgG2b peroxidase (Nordic, the Netherlands) was then added for the enzymatic reaction. TMB (eBioscience) or ABTS (Sigma-Aldrich) was used as a substrate and incubated for an additional 15 minutes before the reaction was stopped with 1 M H_2_SO_4_ and read using a microplate reader (Bio-Rad, the Netherlands) at a wavelength of 450 nm for TMB or 415 nm for ABTS. Cross-reactivity of Abs used in the assay was tested by omitting HLA-A2 from the ELISA.

Alternatively antibody HLA-monomer complexes were made by incubating biotinylated CD89 or MR with biotinylated HLA-A2 monomer and APC-labeled streptavidine (at a ratio of 2 : 2 : 1, resp.) for 15 minutes at room temperature. Monocytes were cultured with the CD89 complex and competition was performed by adding CD89 Ab or MR Ab (as a negative control). Alternatively, moDCs were cultured with the MR complex and MR or CD89 (as a negative control) were added as competing Abs. “Mean fluorescence intensity” (MFI) of the APC-labeled streptavidin was determined by flow cytometry (FACS, BD Biosciences) as a measure for uptake.

### 2.5. Antigen Presentation Assays

Monocytes or moDCs were plated in round bottom 96-well plates (Costar, Cambridge, MA) at a concentration of 3 × 10^5^ cells/well. Ab and monomeric HLA-A2 were conjugated by making use of the high affinity between avidin and biotin: avidinylated-Ab was incubated with biotinylated HLA-A2 at different ratios for 2 h prior to use in experiments. The conjugated mixture (Ab-A2) and biotinylated HLA-A2 alone (A2) were added at different concentrations to the APCs. After 4 h incubation the antigen was washed away and 5 × 10^3^ T-cell clones were added for 24 h incubation. As controls for T-cell specificity, T-cells were cultured with the complex in the absence of APCs and similarly APCs were cultured with the complex in the absence of T-cells. Supernatants were then harvested and measured for IFN-*γ* using an ELISA according to manufacturer's protocol (eBiosciences).

## 3. Results

### 3.1. Differences in Expression of Cell Surface Molecules on Monocytes and moDC

**Figure 1:**
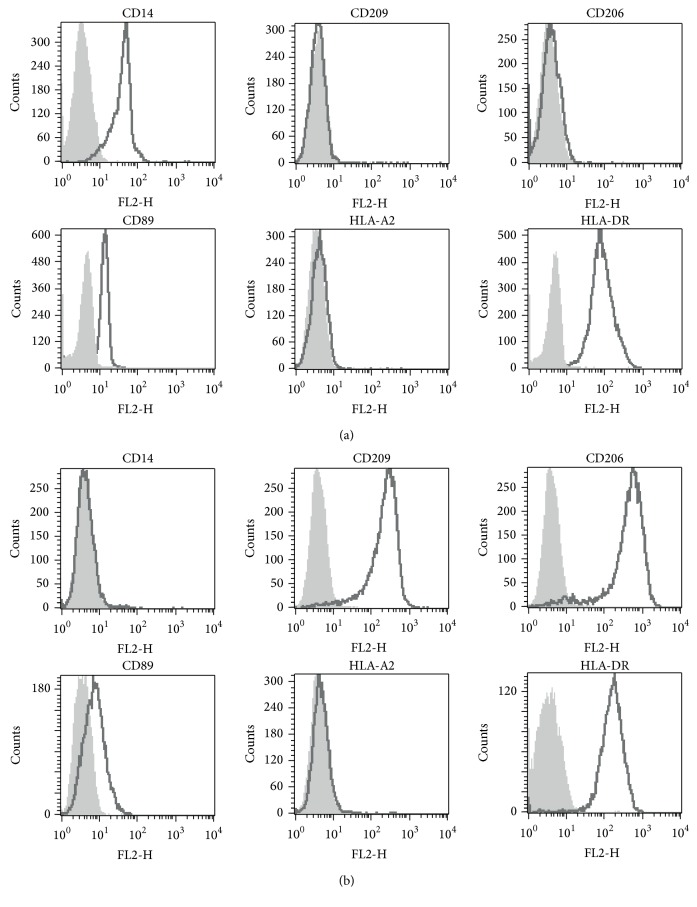
Phenotype and expression of cell surface molecules on monocytes and moDC. Monocytes (a) and moDC (b) were stained with primary antibodies targeting different cell surface molecules. Staining was visualized with goat anti-mouse Ig-F(ab)2-PE. Grey filled histograms depict isotype controls and black bold lined nonfilled histograms depict the staining.

To investigate and identify potential endocytic receptors on monocytes and moDCs an analysis of cell surface molecules was performed (Figure 1). Monocytes expressed high levels of CD14 and CD89 but no detectable levels of “mannose receptor” (MR/CD206) or DC-SIGN (CD209). On the other hand moDCs were negative for CD14 and positive for CD206 and CD209. The endocytic receptor CD89 showed only low expression on moDCs. HLA-DR expression was high on monocytes and even higher on moDC. Monocytes and moDCs were HLA typed for HLA-DR1+ and HLA-A2− so that allopresentation of HLA-A2 restricted by HLA-DR1 could be assessed. Both monocytes and moDC were negative for HLA-A2.

### 3.2. Uptake of the Antibody-Antigen Complex Is Mediated via CD89

**Figure 2:**
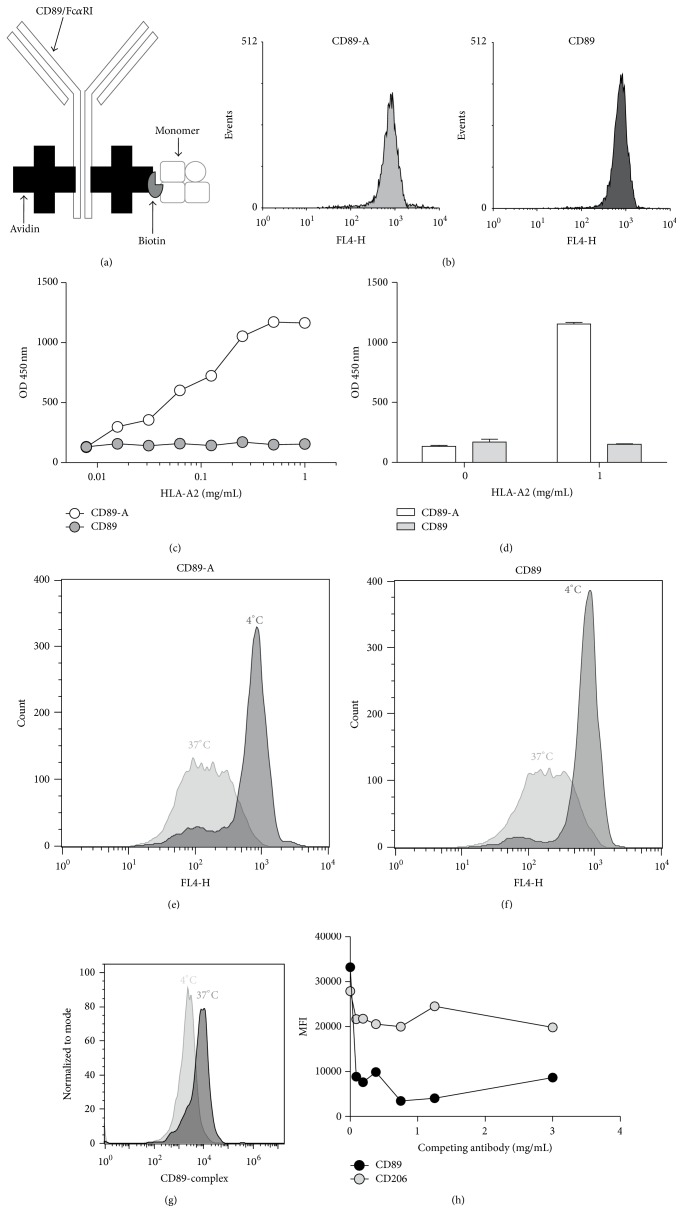
CD89 antibody and HLA-A2 complexes are efficiently bound and taken up by monocytes. (a) Diagram representing the targeting Ab. Positions of conjugated avidin groups depicted in the diagram are arbitrarily chosen. (b) Monocytes were stained with 1 *μ*g/mL avidin conjugated (CD89-A (grey)) or nonconjugated (CD89 (white with black outline)) Ab. Staining was visualized with a secondary APC-labeled Ab. (c) Formation of the CD89-A HLA-A2 immune-complex (CD89-A2) was confirmed by an ELISA conjugated or nonconjugated (CD89-A/CD89) Ab which were coated on a 96-well plate. (d) Ab cross-reactivity was tested by removing HLA-A2 from the system and incubating the antibodies as was done in (c). Grey bars indicate CD89, and white bars are the avidinylated CD89 Ab (CD89-A). ((e) and (f)) To test whether avidinylation had an effect on receptor mediated endocytosis (RME) monocytes were incubated with either conjugated CD89 (CD89-A) or nonconjugated CD89 at 4°C (grey histograms) or at 37°C (white with dark outline histograms). After 2 h incubation CD89 on the cell surface was visualized with a secondary Ab. (g) Uptake was confirmed by formation of a complex with biotinylated-CD89 and HLA-A2 monomer incubated with APC-labeled streptavidine (CD89-A2-strep) at a ratio of 2 : 2 : 1, respectively. The complex was incubated for 24 h at 4°C (light grey) or 37°C (dark grey) in the presence of monocytes. Shown is one representative experiment. (h) To establish CD89 mediated uptake monocytes were cultured with CD89-A2-strep, in the presence of increasing amounts of competing CD89 Ab or MR. After overnight incubation, the APC fluorescence intensity was assessed by flow cytometry as a measure for uptake. Shown is one representative experiment.

We recently described an* in vitro* model using total PBMC populations for the monitoring of indirect presentation [9]. In view of the high expression of CD89 on monocytes, we aimed to target the HLA monomer towards CD89 for more efficient presentation of this alloantigen. Complexes of biotinylated HLA-A2 monomers and the CD89 antibody (2D11) were generated (Figure 2(a)). The 2D11 antibody targeting CD89 was avidinylated (CD89-A) without showing an effect on antibody reactivity; staining of monocytes with CD89-A and CD89 Ab showed similar fluorescence intensity (Figure 2(b)). Validation of the biotinylated HLA-A2 and CD89-A immune-complex was achieved through a specific sandwich ELISA (Figure 2(c)). A dose-dependent binding of biotinylated HLA-A2 could be demonstrated when CD89-A was coated. No response was observed when CD89 was coated, indicating that CD89-A and HLA-A2 (CD89-A-A2) complex was specific. To exclude Ab cross-reactivity, when HLA-A2 was removed from the system, both CD89 and CD89-A showed no reactivity (Figure 2(d)).

Next, we investigated if “receptor mediated endocytosis” (RME) was affected when CD89-A rather than CD89 was used. Therefore, we incubated monocytes with the CD89 or CD89-A Ab for 2 h at 4°C and 37°C (Figures 2(e) and 2(f)). A similar decrease in CD89 expression following 2 h incubation at 37°C was observed, indicating a similar turnover rate for both CD89 and CD89-A Ab. The 4°C condition was used as the steady state control. To establish that entry of the complex was mediated via CD89 we generated a complex of “APC-labeled streptavidine, biotinylated CD89, and biotinylated HLA-A2” (CD89-A2-strep). First, the uptake of the complex was confirmed by incubating the complex with monocytes overnight at either 4 or 37°C (Figure 2(g)). Higher fluorescence intensity was observed after culture at 37°C when compared to 4°C, indicating that measurable uptake was occurring at 37°C. Next, we performed a competition assay by culturing monocytes overnight with CD89-A2-strep and titrating in unlabeled CD89 Ab or an irrelevant Ab (Figure 2(h)). Increasing concentrations of CD89, but not irrelevant mAb (MR), dose-dependently inhibited binding and subsequent uptake of the complex.

### 3.3. CD89 Mediated Uptake of Antigen by Monocytes Does Not Enhance T-Cell Activation When Compared to Antigen Alone

**Figure 3:**
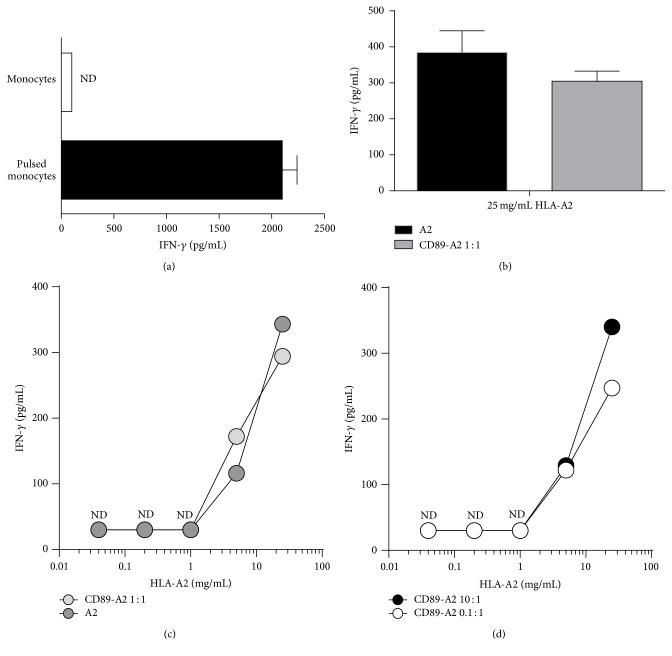
HLA-DR mediated antigen presentation by monocytes is not enhanced through CD89-targeted endocytosis. (a) HLA-DR1+/HLA-A2− monocytes were either pulsed (dark bar) or not (light bar) with HLA-A2 peptides and cocultured with T-cell clone. After 24 h incubation supernatants were harvested and IFN-*γ* was measured. (b) CD89-A was incubated at a ratio of 1 : 1 with biotinylated HLA-A2 monomer for 2 hours (CD89-A2), before they were added to the monocytes for 4 hours and then washed. T-cells were then added and IFN-*γ* measured. In comparison monocytes were also incubated with HLA-A2 alone (A2). ((c) and (d)) Different ratios of CD89 and A2 were used as explained in (b) and titrated. All experiments were performed at least 3 times; shown is the mean with SD of one experiment in triplicate except for (a) and (b) where the mean of 3 experiments in triplicate is shown with SD. ND: nondetectable.

Reactivity of the T-cell clone with indirect specificity was demonstrated by exogenous loading of HLA-DR1+/HLA-A2− monocytes with the specific HLA-A2 peptide (Figure 3(a)). Strong IFN-*γ* production by the T-cell clone was observed in the peptide-pulsed condition only; no reactivity was observed if the monocytes were not pulsed. To investigate the effect of targeting the monomer to CD89 on monocytes, CD89-A-A2 complexes were formed by mixing CD89-A and biotinylated HLA-A2 monomer in a 1 : 1 molar ratio prior to test. Incubation of HLA typed monocytes with both forms of HLA-A2 (25 *μ*g/mL; soluble or as immune-complex) resulted in a similar level of T-cell activation as measured by IFN-*γ* production (Figure 3(b)). To investigate a potential increase in the efficiency of antigen presentation upon CD89 targeting, we tested lower concentrations of monomer. We observed a dose-dependent increase of T-cell reactivity; however none of the conditions showed a more efficient presentation by the CD89-directed complexes (Figure 3(c)). Similarly, the generation of complexes with a different CD89-A-HLA-A2 ratio (0.1 : 1 or 10 : 1) did not result in a more efficient T-cell activation (Figure 3(d)). In conclusion, targeting of the HLA-A2-complexes to CD89 does not lead to an increase in HLA-DR1 mediated presentation by monocytes.

### 3.4. Uptake of the Antibody-Antigen Complex by moDC Is Mediated via CD206

**Figure 4:**
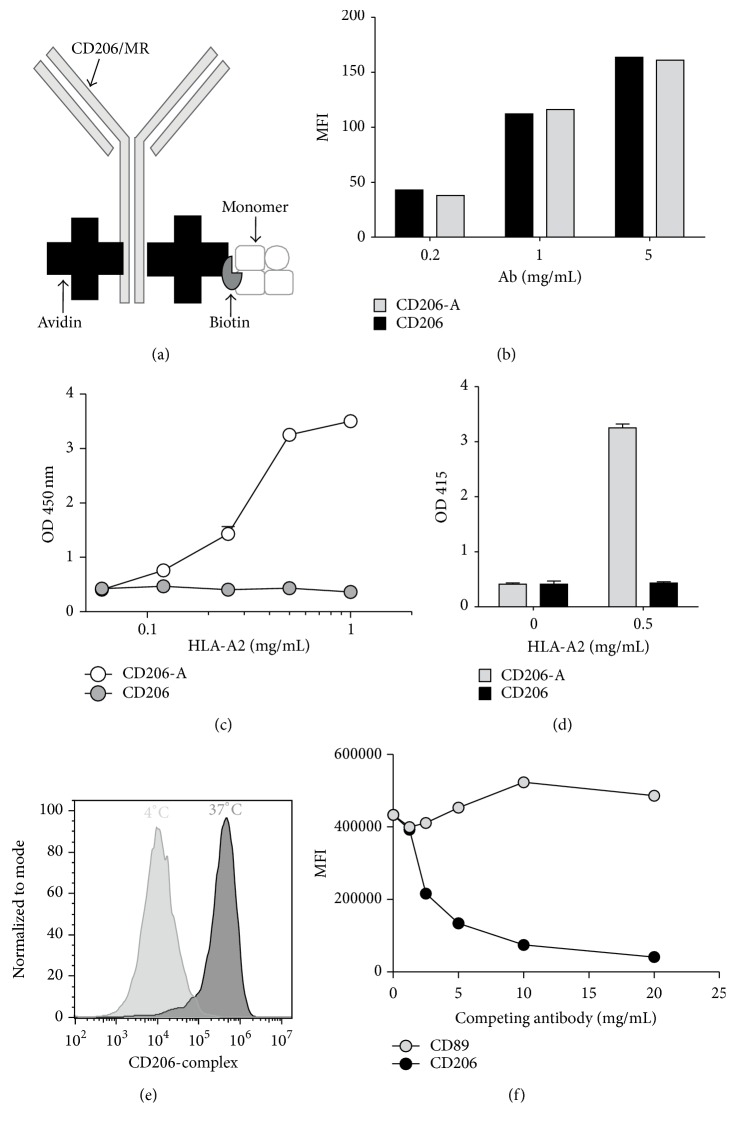
CD206-HLA-A2 complexes are efficiently bound and taken up by moDCs through CD206 receptor mediated endocytosis. (a) Diagrammatic representation of the targeting Ab. Avidin groups conjugated and their position depicted in the diagram are arbitrarily chosen. (b) moDCs were stained with the conjugated (CD206-A) or nonconjugated (CD206) Ab at different concentrations. Indicated is the mean florescence intensity (MFI). (c) Formation of the biotinylated HLA-A2/CD206-A immune-complex was investigated by setting up a sandwich ELISA system as explained in Materials and Methods. Briefly, the CD206/CD206-A was coated onto a 96-well plate, biotinylated HLA-A2 was added at different concentrations, and subsequently an anti-HLA-A2 antibody and a peroxidase targeting the secondary antibody were added. ABTS was used as substrate. (d) Cross-reactivity of the antibodies was ruled out by coating CD206-A (grey bars) or CD206 (dark bars) in the presence or absence of HLA-A2 and incubation of the Ab as depicted in C. CD206-A (grey bars) or CD206 (dark bars) were coated with or without HLA-A2. (e) To demonstrate that CD206-targeted complexes were taken up via CD206, we created complexes of APC-labeled streptavidine, biotinylated HLA-A2 monomer, and CD206 (CD206-A2-strep) at a molecular ratio of, respectively, 1 : 2 : 2. Uptake of CD206-A2-strep was determined by culturing moDC with the complex for 24 h at 4°C (light grey) or 37°C (dark grey). (f) To determine CD206-specific uptake, moDCs were cultured with the CD206-A2-strep complex, with competing MR/CD206 or irrelevant CD89 Ab. After overnight incubation, the APC fluorescence intensity was assessed by flow cytometry. Shown is one representative experiment.

Targeting of antigens via CD206 towards moDCs has been successful in other studies [14, 34, 35]. Therefore we investigated the potential of CD206 to enhance antigen presentation in our experimental model. Immune-complexes of HLA-A2 monomers and antibodies targeting CD206 were created (Figure 4(a)). The CD206 antibody was “avidinylated” (CD206-A) and the binding capacity to moDC was similar to that of the unconjugated Ab (CD206, Figure 4(b)). Dose-dependent formation of the “CD206-A/HLA-A2” (CD206-A-A2) complex was seen when CD206-A Abs were used but not with CD206 (Figures 4(c) and 4(d)). No cross-reactivity was observed, as demonstrated in the condition where no biotinylated monomeric HLA-A2 was used or when unconjugated CD206 was coated (Figure 4(d)). To address whether CD206-targeted complexes are taken up via CD206, we created a complex of APC-labeled streptavidin with biotinylated “CD206 and HLA-A2” (CD206-A2-strep). Uptake of CD206-A2-strep was confirmed by incubating the complex with moDC overnight at either 4 or 37°C (Figure 4(e)). The fluorescence intensity was considerably higher at 37°C, as compared to 4°C, showing that the CD206-A2-strep is taken up. Next, we performed a competition assay by incubating CD206-A2-strep with moDC overnight, in the presence of increasing amounts of CD206 (MR) Ab or a control Ab (CD89), to confirm CD206-specific endocytosis (Figure 4(f)). Increasing concentrations of the CD206 Ab inhibited binding and subsequent uptake of the complex in a dose-dependent manner, whereas increasing concentrations of the irrelevant Ab did not affect the uptake of the complex.

### 3.5. CD206 Mediated Uptake of Antigen by Monocytes Does Not Enhance T-Cell Activation When Compared to Antigen Alone

**Figure 5:**
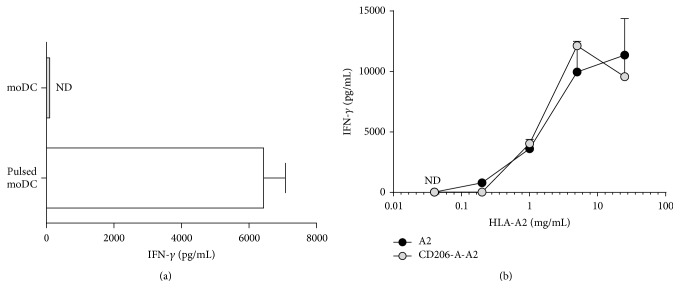
Antigen presentation is not improved by CD206 mediated endocytosis. (a) T-cell clone specificity was confirmed by incubating the T-cell clones with HLA-DR1+/HLA-A2− moDCs that were either pulsed or not pulsed with HLA-A2 derived peptides. (b) DR1+/A2− moDCs were incubated with different concentrations of monomeric HLA-A2 (A2) or the CD206-A HLA-A2 immune-complex (CD206-A-A2 ratio 3 : 1). T-cells were added to the culture following 4 h incubation with the antigen and subsequent washing steps. IFN-*γ* was measured in the supernatants after 24 h. Each experiment was conducted at least 3 times; shown is the mean of one experiment (performed in triplicate) with SD. ND: nondetectable.

The reactivity of the T-cell clone with indirect specificity showed a potent IFN-*γ* response exclusively when moDCs were pulsed (Figure 5(a)). The response observed with moDCs was higher than when monocytes were used. Both the CD206-A-A2 complex and HLA-A2 monomer gave a dose-dependent increase of T-cell activation (Figure 5(b)). However, no difference in efficiency of Ag presentation was observed when HLA monomer alone was compared to the CD206-A-A2 condition. Other ratios of monomer and antibody led to similar results (data not shown). In conclusion, targeting of the HLA-A2-complexes to CD206 does not lead to an increase in HLA-DR1 mediated presentation by moDCs.

## 4. Discussion

In an effort to measure indirect allorecognition in transplant recipients we have recently developed a method utilizing HLA monomers [9]. In the present study we tried to improve the efficiency of this assay by enhancing uptake of HLA class I antigens via targeting of endocytic receptors on either monocytes or moDCs. Targeting of HLA-A2 to CD89 on monocytes and CD206 on moDCs led to efficient antigen delivery into the cell and presentation of the relevant allopeptide to a CD4 T-cell clone. However, under these conditions antigen targeting did not enhance the efficiency of antigen presentation when compared to soluble HLA-A2 alone.

The human IgA Fc receptor (CD89) is expressed on various myeloid cells and has been previously shown to mediate antigen uptake, processing, and even presentation of defined antigens [15, 17, 19–21]. Cross-linking of CD89 on B-cells transfected with Fc*α*RI activates PI3-kinase/phosphatidyl inositol-dependent kinase 1/PKB*α* signaling pathway and inhibition of PI3-kinase blocks MHC class II presentation of CD89-targeted antigen [15]. The presentation of CD89-targeted antigen was found to be enhanced by *γ*-chain signaling which contains an “immunoreceptor tyrosine-based activation motif” (ITAM) [20]. Interestingly, this *γ*-chain signaling has recently been implicated in CD4 T-cell priming and MHC class II recycling [36]. However, antigen presentation of CD89-targeted cargo on moDCs did not lead to efficient antigen presentation [37], possibly due to the low expression of CD89 on moDC, as we previously demonstrated [18]. Still this leaves the question why we did not see an enhanced presentation and T-cell activation when HLA monomers were targeted towards CD89. One explanation might be that uptake through CD89 results in a different trafficking and preferential degradation in monocytes. CD89-mediated presentation relies heavily on trafficking by protein kinases. Two distinct studies have shown that protein kinase B and protein kinase C *α* and *δ* mediate CD89 trafficking and subsequently inhibit presentation [21, 38]. As both studies were conducted in transfected B-cells it is possible that trafficking in monocytes is different.

One other point of concern is the expression of CD89 on monocytes from patients undergoing immunosuppressive therapy. It is known that CD89 expression can be downregulated by TGF-*β*1 and by soluble IgA in patients with IgA nephropathy [39, 40]. Whether CD89 is downregulated in transplant patients undergoing immunosuppressive therapy remains to be established.

As an alternative strategy of an APC, we opted for the use of the professional APC moDCs and identified MR expression as a receptor for targeting. The role of the MR in mediating immune-responses has been previously reviewed [24, 25]. The first studies into the MR showed its capacity to endocytose cargo [41]. It is this capacity that has led to recent studies using sulfated carbohydrates [22] and antibody-antigen immune-complexes [23, 26, 42] for antigen endocytosis and presentation. It is clear that targeting of antigens to the MR results in efficient presentation via MHC class II and CD4 priming [14, 23, 27]. Interestingly when mannosylated peptides or proteins were used a 1000-fold increase in HLA class II mediated antigen presentation was seen when compared to nonmannosylated antigens [14, 34]. However, it has now been established that mannosylated antigens can bind to multiple CLRs including MR and DC-SIGN, which are both expressed on moDCs. Studies into endocytosis via DC-SIGN observed MHC class II mediated antigen presentation [43], although more recent studies showed that DC-SIGN targeted cargo can also be cross-presented to CD8 T-cells [44].

In our experiments CD206 targeted immune-complexes were endocytosed and subsequently presented via HLA-DR1. However, the presentation was not enhanced when compared with soluble antigen. As the immune-complex is taken up, it is possible that peptides derived from the Ab are also presented and compete with the HLA-A2-derived peptide. In addition, there are studies that have failed to show improvement in HLA class II mediated antigen presentation, following MR-mediated antigen presentation [23, 45]. Interestingly, when Tsuji and colleagues used Ab-antigen fusion proteins targeting the MR, they found no enhancement in HLA class II mediated presentation when compared to soluble antigen but rather found HLA class I-mediated presentation [23]. This indicates that MR-mediated endocytosis leads to cross-presentation of the antigenic cargo, which was further corroborated by other groups [23, 26, 46]. It is possible that this also occurs in our experimental model. A major point that makes these studies difficult to compare is that in all cases different antigens and different Abs were used. Therefore it is possible that different processing mechanisms are involved, which may explain some of the observed discrepancies.

Donor alloantibodies are frequently found in rejected grafts and play an important role in allograft rejection [47]. Multiple studies have shown that about 20% of patients develop alloantibodies, despite immunosuppressive therapy [48, 49]. It is likely that formation of immune-complexes between alloantibodies and HLA monomers, used within the assay, can affect uptake and presentation. However, since the assay can be performed with purified PBMC, in the absence of serum, this might be less of a problem.

Despite the absence of improved efficiency of antigen loading and presentation* in vitro*, both targets could still prove to be good candidates for antigenic targeting to monocytes through CD89 and to DCs through CD206* in vivo*. In such a way, targeting of antigens to endocytic receptors might still have an important impact on the induction of transplant tolerance as demonstrated by Tanriver et al. In an experimental model targeting of MHC class I monomers/Ab complexes to 33D1 on DCs led to inhibition of indirect allorecognition and the abrogation of alloantibody production leading to graft survival [50]. This is further supported by* in vivo* studies where antigen targeting via DEC-205 led to tolerance induction, as the maturation of the DCs was not affected by this procedure [51]. This could also be the case for CD206 targeting and might be a useful tool to target antigens for tolerance induction. In view of these different biological activities, it will be of importance to study in detail antigen presentation properties of the different members of the C-type lectin family but also to include the specific properties of the antigens used for targeting.

In conclusion our data demonstrate that targeting of antigens to CD206 on moDCs and CD89 on monocytes can lead to antigen processing and presentation via HLA-DR to T-cells, in a specific and dose-dependent fashion. However, we also demonstrate that although the complex is internalized and presented, there is no improvement in antigen presentation when compared to soluble antigen and thus it does not enhance indirect allopresentation.

## Abbreviations

APC: Antigen presenting cells
CLR: C-type lectin receptor
MFI: Mean fluorescence intensity
MR: Mannose receptor
moDC: Monocytes-derived dendritic cells
RME: Receptor mediated endocytosis.

## References

[1] Ali J. M., Bolton E. M., Bradley J. A., Pettigrew G. J. (2013). Allorecognition pathways in transplant rejection and tolerance. *Transplantation*.

[2] Conlon T. M., Saeb-Parsy K., Cole J. L. (2012). Germinal center alloantibody responses are mediated exclusively by indirect-pathway CD4 T follicular helper cells. *Journal of Immunology*.

[3] Gaughan A., Wang J., Pelletier R. P. (2014). Key role for CD4 T cells during mixed antibody-mediated rejection of renal allografts. *American Journal of Transplantation*.

[4] Lee P.-C., Ozawa M., Hung C.-J., Lin Y.-J., Chang S.-S., Chou T.-C. (2009). Eighteen-year follow-up of a retrospective study of HLA antibody on kidney graft survival. *Transplantation Proceedings*.

[5] Bestard O., Nickel P., Cruzado J. M. (2008). Circulating alloreactive T cells correlate with graft function in longstanding renal transplant recipients. *Journal of the American Society of Nephrology*.

[6] Lamb K. E., Lodhi S., Meier-Kriesche H.-U. (2011). Long-term renal allograft survival in the United States: a critical reappraisal. *American Journal of Transplantation*.

[7] Lodhi S. A., Lamb K. E., Meier-Kriesche H. U. (2011). Solid organ allograft survival improvement in the United States: the long-term does not mirror the dramatic short-term success. *American Journal of Transplantation*.

[8] Waanders M. M., Heidt S., Koekkoek K. M. (2008). Monitoring of indirect allorecognition: wishful thinking or solid data?. *Tissue Antigens*.

[9] Breman E., van Miert P. P., van der Steen D. M. (2014). HLA monomers as a tool to monitor indirect allorecognition. *Transplantation*.

[10] Neefjes J., Jongsma M. L. M., Paul P., Bakke O. (2011). Towards a systems understanding of MHC class I and MHC class II antigen presentation. *Nature Reviews Immunology*.

[11] Joffre O. P., Segura E., Savina A., Amigorena S. (2012). Cross-presentation by dendritic cells. *Nature Reviews Immunology*.

[12] Burgdorf S., Kurts C. (2008). Endocytosis mechanisms and the cell biology of antigen presentation. *Current Opinion in Immunology*.

[13] Mahnke K., Guo M., Lee S. (2000). The dendritic cell receptor for endocytosis, DEC-205, can recycle and enhance antigen presentation via major histocompatibility complex class II-positive lysosomal compartments. *Journal of Cell Biology*.

[14] Tan M. C. A. A., Mommaas A. M., Drijfhout J. W. (1997). Mannose receptor-mediated uptake of antigens strongly enhances HLA class II-restricted antigen presentation by cultured dendritic cells. *European Journal of Immunology*.

[15] Lang M. L., Shen L., Gao H., Cusack W. F., Lang G. A., Wade W. F. (2001). Fc*α* receptor cross-linking causes translocation of phosphatidylinositol-dependent protein kinase 1 and protein kinase B*α* to MHC class II peptide-loading-like compartments. *Journal of Immunology*.

[16] Birkholz K., Schwenkert M., Kellner C. (2010). Targeting of DEC-205 on human dendritic cells results in efficient MHC class II-restricted antigen presentation. *Blood*.

[17] Pasquier B., Lepelletier Y., Baude C., Hermine O., Monteiro R. C. (2004). Differential expression and function of IgA receptors (CD89 and CD71) during maturation of dendritic cells. *Journal of Leukocyte Biology*.

[18] Heystek H. C., Moulon C., Woltman A. M., Garonne P., Van Kooten C. (2002). Human immature dendritic cells efficiently bind and take up secretory IgA without the induction of maturation. *Journal of Immunology*.

[19] Tacken P. J., Batenburg J. J. (2006). Monocyte CD64 or CD89 targeting by surfactant protein D/anti-Fc receptor mediates bacterial uptake. *Immunology*.

[20] Shen L., Van Egmond M., Siemasko K. (2001). Presentation of ovalbumin internalized via the immunoglobulin-A Fc receptor is enhanced through Fc receptor *γ*-chain signaling. *Blood*.

[21] Chen Y.-W., Lang M. L., Wade W. F. (2004). Protein kinase C-*α* and -*δ* are required for Fc*α*R (CD89) trafficking to MHC class II compartments and Fc*α*R-mediated antigen presentation. *Traffic*.

[22] Singh S. K., Streng-Ouwehand I., Litjens M. (2011). Design of neo-glycoconjugates that target the mannose receptor and enhance TLR-independent cross-presentation and Th1 polarization. *European Journal of Immunology*.

[23] Tsuji T., Matsuzaki J., Kelly M. P. (2011). Antibody-targeted NY-ESO-1 to mannose receptor or DEC-205 in vitro elicits dual human CD^8+^ and CD^4+^ T cell responses with broad antigen specificity. *Journal of Immunology*.

[24] Gazi U., Martinez-Pomares L. (2009). Influence of the mannose receptor in host immune responses. *Immunobiology*.

[25] Martinez-Pomares L. (2012). The mannose receptor. *Journal of Leukocyte Biology*.

[26] Chatterjee B., Smed-Sörensen A., Cohn L. (2012). Internalization and endosomal degradation of receptor-bound antigens regulate the efficiency of cross presentation by human dendritic cells. *Blood*.

[27] Ramakrishna V., Treml J. F., Vitale L. (2004). Mannose receptor targeting of tumor antigen pmel17 to human dendritic cells directs anti-melanoma T cell responses via multiple HLA molecules. *The Journal of Immunology*.

[28] He L.-Z., Crocker A., Lee J. (2007). Antigenic targeting of the human mannose receptor induces tumor immunity. *Journal of Immunology*.

[29] Woltman A. M., de Fijter J. W., Kamerling S. W. A., Paul L. C., Daha M. R., Van Kooten C. (2000). The effect of calcineurin inhibitors and corticosteroids on the differentiation of human dendritic cells. *European Journal of Immunology*.

[30] Uccini S., Sirianni M. C., Vincenzi L. (1997). Kaposi's sarcoma cells express the macrophage-associated antigen mannose receptor and develop in peripheral blood cultures of Kaposi's sarcoma patients. *The American Journal of Pathology*.

[31] Morton H. C., van Zandbergen G., van Kooten C., Howard C. J., van de Winkel J. G. J., Brandtzaeg P. (1999). Immunoglobulin-binding sites of human Fc*α*RI (CD89) and bovine Fc*γ*2R are located in their membrane-distal extracellular domains. *The Journal of Experimental Medicine*.

[32] Amir A. L., Hagedoorn R. S., van Luxemburg-Heijs S. A. P. (2012). Identification of a coordinated CD8 and CD4 T cell response directed against mismatched HLA class I causing severe acute graft-versus-host disease. *Biology of Blood and Marrow Transplantation*.

[33] Altman J. D., Moss P. A., Goulder P. J. (1996). Phenotypic analysis of antigen-specific T lymphocytes. *Science*.

[34] Engering A. J., Cella M., Fluitsma D. (1997). The mannose receptor functions as a high capacity and broad specificity antigen receptor in human dendritic cells. *European Journal of Immunology*.

[35] Loveland B. E., Zhao A., White S. (2006). Mannan-MUC1—pulsed dendritic cell immunotherapy: a phase I trial in patients with adenocarcinoma. *Clinical Cancer Research*.

[36] Graham D. B., Akilesh H. M., Gmyrek G. B. (2010). ITAM signaling in dendritic cells controls T helper cell priming by regulating MHC class II recycling. *Blood*.

[37] Otten M. A., Groenveld I., van de Winkel J. G. J., van Egmond M. (2006). Inefficient antigen presentation via the IgA Fc receptor (Fc*α*RI) on dendritic cells. *Immunobiology*.

[38] Lang G. A., Lang M. L. (2006). Protein kinase B*α* is required for vesicle trafficking and class II presentation of IgA Fc receptor (CD89)-targeted antigen. *Journal of Immunology*.

[39] Reterink T. J. F., Levarht E. W. N., Klar-Mohamad N., Van Es L. A., Daha M. R. (1996). Transforming growth factor-beta 1 (TGF-*β*1) down-regulates IgA Fc-receptor (CD89) expression on human monocytes. *Clinical and Experimental Immunology*.

[40] Grossetête B., Launay P., Lehuen A., Jungers P., Bach J.-F., Monteiro R. C. (1998). Down-regulation of Fc*α* receptors on blood cells of IgA nephropathy patients: evidence for a negative regulatory role of serum IgA. *Kidney International*.

[41] Robbins J. C., Lam M. H., Tripp C. S., Bugianesi R. L., Ponpipom M. M., Shen T. Y. (1981). Synthetic glycopeptide substrates for receptor-mediated endocytosis by macrophages. *Proceedings of the National Academy of Sciences of the United States of America*.

[42] Morse M. A., Chapman R., Powderly J. (2011). Phase I study utilizing a novel antigen-presenting cell-targeted vaccine with toll-like receptor stimulation to induce immunity to self-antigens in cancer patients. *Clinical Cancer Research*.

[43] Engering A., Geijtenbeek T. B. H., van Vliet S. J. (2002). The dendritic cell-specific adhesion receptor DC-SIGN internalizes antigen for presentation to T cells. *The Journal of Immunology*.

[44] Tacken P. J., Ginter W., Berod L. (2011). Targeting DC-SIGN via its neck region leads to prolonged antigen residence in early endosomes, delayed lysosomal degradation, and cross-presentation. *Blood*.

[45] Napper C. E., Taylor M. E. (2004). The mannose receptor fails to enhance processing and presentation of a glycoprotein antigen in transfected fibroblasts. *Glycobiology*.

[46] Burgdorf S., Schuette V., Semmling V. (2010). Steady-state cross-presentation of OVA is mannose receptor-dependent but inhibitable by collagen fragments. *Proceedings of the National Academy of Sciences of the United States of America*.

[47] Mehra N. K., Siddiqui J., Baranwal A., Goswami S., Kaur G. (2013). Clinical relevance of antibody development in renal transplantation. *Annals of the New York Academy of Sciences*.

[48] Terasaki P. I., Ozawa M. (2004). Predicting kidney graft failure by HLA antibodies: a prospective trial. *American Journal of Transplantation*.

[49] Terasaki P. I., Ozawa M., Castro R. (2007). Four-year follow-up of a prospective trial of HLA and MICA antibodies on kidney graft survival. *American Journal of Transplantation*.

[50] Tanriver Y., Ratnasothy K., Bucy R. P., Lombardi G., Lechler R. (2010). Targeting MHC class I monomers to dendritic cells inhibits the indirect pathway of allorecognition and the production of IgG alloantibodies leading to long-term allograft survival. *Journal of Immunology*.

[51] Hawiger D., Inaba K., Dorsett Y. (2001). Dendritic cells induce peripheral T cell unresponsiveness under steady state conditions in vivo. *The Journal of Experimental Medicine*.

